# The Evolution of Mindfulness-Based Physical Interventions in Breast Cancer Survivors

**DOI:** 10.1155/2012/758641

**Published:** 2012-09-11

**Authors:** Daniela L. Stan, Nerissa M. Collins, Molly M. Olsen, Ivana Croghan, Sandhya Pruthi

**Affiliations:** ^1^Department of General Internal Medicine, Mayo Clinic, 200 First Street SW, Rochester, MN 55905, USA; ^2^Nicotine Research Program, Primary Care Internal Medicine, Mayo Clinic, 200 First Street SW, Rochester, MN 55905, USA

## Abstract

Survivors of breast cancer are faced with a multitude of medical and psychological impairments during and after treatment and throughout their lifespan. Physical exercise has been shown to improve survival and recurrence in this population. Mind-body interventions combine a light-moderate intensity physical exercise with mindfulness, thus having the potential to improve both physical and psychological sequelae of breast cancer treatments. We conducted a review of mindfulness-based physical exercise interventions which included yoga, tai chi chuan, Pilates, and qigong, in breast cancer survivors. Among the mindfulness-based interventions, yoga was significantly more studied in this population as compared to tai chi chuan, Pilates, and qigong. The participants and the outcomes of the majority of the studies reviewed were heterogeneous, and the population included was generally not selected for symptoms. Yoga was shown to improve fatigue in a few methodologically strong studies, providing reasonable evidence for benefit in this population. Improvements were also seen in sleep, anxiety, depression, distress, quality of life, and postchemotherapy nausea and vomiting in the yoga studies. Tai chi chuan, Pilates, and qigong were not studied sufficiently in breast cancer survivors in order to be implemented in clinical practice.

## 1. Introduction

Cancer survivorship, as a distinct and complex phase of the cancer journey, has gained strong support by both survivors of breast cancer and their clinicians. Breast cancer survivors account for 2.4 million of the 10 million cancer survivors in the United States [1].

Survivors of breast cancer are faced with a multitude of medical and psychological impairments during and after treatment and throughout their lifespan. One intervention shown convincingly to improve some of the long-term toxicities and late effects of therapy is physical exercise [2, 3]. Moderate physical exercise has also been associated with reducing breast cancer risk among postmenopausal women [4]. Aerobic training has also been shown to increase recurrence-free and overall survival in this population [5]. Such conventional exercise regimens however can be challenging for women who have undergone surgery, chemotherapy, and/or radiation treatment who are also experiencing the additional burden of anxiety and stress about the possibility of recurrence.

Thus, it is not surprising that breast cancer survivors are increasingly exploring interventions that combine mind and body components, herein referred to as “mindful exercise.” Mindful exercise includes yoga, tai chi chuan, Pilates, and qigong. These exercise interventions address both physical and psychological needs simultaneously and may be particularly appealing to breast cancer survivors [6]. A growing number of research studies in breast cancer survivors have demonstrated beneficial effects such as improving quality of life (QOL) and fitness levels while reducing fatigue and depression [7–10]. 

Published reviews of mindful exercise interventions lack the comprehensive inclusiveness that is part of the mindful-based exercise modalities (yoga, tai chi chuan, Pilates, and qigong) but instead focus on only one method. These interventions are similar in their philosophy of affecting both the mind and the body, and they target similar outcomes of QOL, mood, and fatigue. In this paper, we provide a comprehensive review of the scientific evidence on the effect of all mindful exercise interventions in breast cancer survivors. 

## 2. Background

Heightened awareness of cancer survivorship issues in the late 1990s, coupled with evidence that most cancer survivors use one or more forms of complementary and alternative medicine (CAM) [11], led to an increased recognition of the need for rigorous research in this arena. The Office of Cancer Complementary and Alternative Medicine and the National Center for Complementary and Alternative Medicine, created within the National Cancer Institute, encourage and support the scientific scrutiny of CAM therapies for treatment of cancer and cancer sequelae. This growing body of research has led to a renaissance in how both patients and physicians address the wellness of the whole person, including the physical, psychological, and spiritual needs.

A high proportion of breast cancer survivors use one or more types of CAM, the most popular categories being the “natural products” and “mind-body practices” [6, 12, 13]. Mind-body practices target the “interaction between brain, mind, body, and behavior, with the intent to use the mind to affect the physical function and promote health” [14]. These practices include meditation, guided imagery, deep-breathing exercises, progressive relaxation, hypnotherapy, yoga, tai chi chuan, qigong, and acupuncture. Some of these methods are mostly meditative, while others such as yoga, tai chi chuan, and qigong include a significant physical component which qualifies them as distinct fitness methods. Another fitness method that is part of the “movement” category of CAM therapies is the Pilates method [14]. 

In the general population, yoga and Pilates are the fastest growing fitness methods [15]. These interventions are greatly advertised to breast cancer survivors as effective methods of rehabilitation, despite limited scientific evidence of their benefit on QOL and other psychological parameters. It has been claimed that the mindfulness component connects the body, mind, spirit, thus having beneficial effects on depression, anxiety, fatigue, and pain [16–18]. However, the majority of the research on mind-body interventions involves studies that are small and lack a proper comparison group [19–21].

Given the importance of physical activity in improving sequelae of breast cancer treatment and decreasing the recurrence and mortality rates, it is prudent that clinicians and researchers understand if physical exercise with a mindful component has similar effects. The purpose of our paper is to provide an overview of the scientific evidence of the effect of all mindful exercise interventions among breast cancer survivors. 

## 3. Yoga

The most studied of the mindful exercise interventions, yoga, has origins in the Buddhist tradition about 5000 years ago. There are 13 types of yoga, some more meditative (Kundalini) and others more physical (Hatha and the styles derived from it: Iyengar, Ashtanga, and Vinyasa) [22], but they all involve the union between mind, body, and spirit. 

The most practiced in the Western world today is Hatha yoga and its derivations. A survey conducted in 2007 by the National Center for Complementary and Alternative Medicine on the use of CAM by Americans demonstrated that more than 13 million adults had used yoga in the previous year and that the use of yoga among adults increased by 3million people between 2002 and 2007. According to this survey, the most common reasons people use yoga for are its beneficial effects on anxiety, stress, asthma, high blood pressure, and depression, but yoga is also used as part of a general health regimen for physical fitness and relaxation [23]. 

Hatha yoga includes a vast array of asanas (postures done with awareness) and pranayama (regulated breathing through the nostrils), with the purpose of relaxing the body and quieting the thoughts [18, 19]. Iyengar yoga focuses on alignment and holding poses, while the Ashtanga yoga features poses that flow together [22]. Scientific inquiries of yoga benefits started to occur in the early 1970s with reports of benefits in medical ailments such as hypertension, anxiety, depression, and back pain, to name a few. Over the last decade, the number of studies addressing Hatha yoga in cancer survivors has surged and their methodologic quality is improving.

## 4. Tai Chi Chuan (TCC)

Tai chi chuan is a multicomponent intervention that has its origins in China, as a martial art. It combines meditation, graceful movement, deep breathing, and relaxation to move vital energy throughout the body [49]. Over the past 20 years, TCC has been found to be successful at reducing falls and improving sleep in the elderly [50, 51], improving QOL and increasing exercise tolerance in heart failure patients [49, 52], improving physical functioning in patients with rheumatoid arthritis [53], improving blood pressure and cholesterol in hypertensive patients [8], and improving bone mineral density [54]. Recent methodologically stronger studies have brought TCC to the attention of the medical community by demonstrating significant improvements in symptoms and QOL in patients with fibromyalgia [55] and in the balance, functional capacity, and falls risk of patients with Parkinson's disease [56]. In cancer populations, tai chi chuan was studied mostly in breast cancer survivors. 

## 5. Pilates

The Pilates method was developed by the German trainer Joseph Pilates in the 1930s. It combines exercises inspired from yoga, karate, Zen meditation, and the ancient Greek and Roman philosophies of achieving physical and mental perfection [57, 58]. Pilates strengthens the core muscles which subsequently can lead to improvement in spine flexibility and posture [59]. Initially popular with professional dancers and later adopted by professional athletes, Pilates has become extremely popular in the general population. The practice of Pilates has increased by 450% since 2000, with 8.6 million participants in 2009 [15]. The medical field also followed suit, with more than fifty peer-reviewed publications reporting beneficial effects from Pilates in health-related conditions such as back pain, orthopedic rehabilitation, fibromyalgia, and QOL in the elderly [60–63]. 

Pilates exercises are practiced on a floor mat, on a special Pilates chair, or with the help of a spring machine (the Pilates reformer). While performing the exercises, the awareness is on breathing and muscle control. Positive claims associated with Pilates include increased flexibility, range of motion (ROM), muscle endurance, cardiorespiratory fitness, mood level, motivation, energy level, and coordination. However, many of these claims are poorly supported with evidence-based studies [64]. 

## 6. Qigong

This is a form of Chinese health exercise and is an important part of Chinese traditional medicine, consisting of a combination of slow movements, self-massage, slow breathing, healing posture, and meditation [7, 18]. It is the most commonly practiced form of mindful exercise used worldwide, having been practiced for over 3,000 years. Qigong has been studied intensively in medical conditions including depression, hypertension, cardiovascular disease, and anxiety. Studies of qigong in cancer survivors (*N* = 15 studies) have reported benefits in QOL, mood, fatigue, and inflammation [72]. 

## 7. Methods

Two independent reviewers performed a literature search of the Ovid databases (EMBASE and MEDLINE) from inception until February 2012 (in the title area) and the PubMed database (in the title/abstract area) for the following terms: “yoga AND breast cancer,” “tai chi chuan AND breast cancer,” “Pilates AND breast cancer,” “qigong AND breast cancer”, and “mindful exercise AND breast cancer.” Included in this analysis were the following types of human studies: randomized controlled (RCT), nonrandomized controlled (CCT), one-arm pilot studies, and surveys. Excluded from the analysis were reviews (systematic and nonsystematic), case reports, case series, and conference abstracts. 

One investigator (DLS) then assessed the results and excluded studies in which the mindful exercise intervention was not specifically targeted to cancer survivors and those that were obvious duplicates. The reference list of the studies included was evaluated for missed publications and then these were included in the study. Studies for which the outcomes were reported as two to four separate publications were combined in a single entry. 

The articles that met the inclusion criteria were reviewed independently by two investigators and the relevant data were abstracted. 

## 8. Results

**Yoga**

The search “yoga AND breast cancer” identified 42 publications, of which 25 met the inclusion criteria. These included 17 RCTs, 1 survey, and 7 one-arm pilot studies.  Table 1 describes the study design, type of yoga studied, outcome measured, and results of these studies.

**Tai Chi Chuan**

The search “tai chi chuan AND breast cancer” identified 11 studies of TCC in breast cancer survivors, and 5 qualified for inclusion: 4 RCTs and 1 one-arm pilot study, as described in Table 2.

**Pilates**

The search “Pilates AND breast cancer” identified 3 studies addressing the effect of the Pilates exercises in breast cancer survivors. Two were included in this review (1 RCT and 1 one-arm pilot). These studies are described in Table 3.

**Qigong**

The search “qigong AND breast cancer” identified 5 studies of qigong in breast cancer survivors and 4 were included in the analysis: 1 RCT, 2 CCTs, and 1 one-arm pilot, as listed in Table 4.

## 9. Discussion

This comprehensive review reveals that yoga is the most studied of the mindful exercise interventions in breast cancer survivors, whereas TCC, Pilates, and qigong are less well represented.

**Yoga**

Historically, the first publication of the effects of yoga in breast cancer survivors appeared in 2003 [75]. In this study, yoga was part of a more comprehensive intervention named “Mindfulness-Based Stress Reduction” (MBSR) in a sample of breast cancer (*N* = 59) and prostate cancer (*N* = 10) survivors. There was evidence of increased QOL and sleep quality and decreased stress after the intervention, but without a control group, the findings are limited.

It was not until 2006, when a survey of 2022 survivors of any cancer in the Nurses Health Study [6] showed that 62% of this population used one or more CAM methods, that the interest in yoga use in cancer survivorship increased. In this study, yoga was the only CAM intervention that increased the QOL compared to the nonusers of CAM. In fact, users of CAM methods other than yoga had a lower QOL compared to the nonusers in this study, a finding also seen in a previous study of general cancer survivors [76]. 

We identified 24 studies of yoga in breast cancer survivors. The outcomes assessed in these studies are heterogeneous, although some of them are a recurrent theme, such as fatigue (9 trials, 6 showing significantly favorable results), QOL (8 trials, all positive), anxiety (8 studies, all positive), and depression (9 studies, 8 positive). Other less common outcomes assessed were sleep (5 studies, 2 positive), stress, mood, mental health, affect, spirituality, vitality, distress, pain, physical fitness, cognition, chemo-induced nausea, and vomiting. Statistical significant or trends toward improvements were shown for all of these outcomes in the majority of the studies reviewed here. Notable negative results were found for weight, BMI, and hip circumference in a study of postmenopausal obese or overweight breast cancer survivors [28]. In this study, despite a slight weight gain, there was a significant decrease in the waist circumference of −3.1 cm in the yoga versus control population.

The population selected for these studies was relatively heterogeneous with respect to stage of disease and time since breast cancer diagnosis. In addition, the participants were generally not selected for a medical condition or symptom. Barton and Pachman [80] recommended that trials of mind-body interventions should include symptomatic patients in order for an effect size attributable to the intervention to be measurable. This might explain the heterogeneity of effect sizes observed in a systematic review of the effect of yoga on psychological outcomes in cancer survivors (mostly breast cancer) [81]. This review concluded that, although evidence for benefits exists, these should be interpreted with caution given the methodological flaws of the studies. 

One notable exception is a study by Bower et al. [17] that selected a homogeneous population of fatigued (scores of ≤50 on the SF-36 vitality scale) stage 0-II breast cancer survivors. There was significant improvement in the fatigue level in yoga versus control wait-list intervention with a large effect size (*d* = 1.5), superior to other behavioral interventions is managing fatigue in cancer patients [82]. 

Fatigue is one of the most commonly assessed outcomes in the studies reviewed. Indeed, one-third of patients with cancer report persistent fatigue at five to ten years from diagnosis [83] and no interventions were clearly shown to improve or decrease the duration of this symptom. Yoga seems to be promising in this regard. A recent systematic review [84] addressing the effects of a yoga intervention on fatigue in breast cancer survivors showed improvement in fatigue scores (SMD = 0.33, CI 0.01–0.65, *P* = 0.04). In fatigued patients, a low-moderate intensity physical exercise such as yoga might be more appealing and feasible than the regular aerobic exercises. Indeed, in the study by Bower et al. [17] the adherence to the intervention was excellent (80%), much higher compared to aerobic exercise intervention studies [2]. Furthermore, the program evaluation was excellent in all the studies assessing this outcome, suggesting that yoga is a popular intervention in breast cancer patients. 

The lack of an active control group further impacts the methodology of these studies. Only 3 out of 18 RCTs had an active control group of physical activity intervention, whereas 15 were controlled with brief supportive therapy or wait list. Without a control group undergoing a nonmindfulness exercise intervention, it is very challenging to differentiate whether the benefits observed are specific to the yoga intervention or could be attributed to any exercise method or simply to the attention bias (for the studies with a wait-list design). Indeed, a recent systematic review comparing mindfulness-based exercise versus nonmindfulness exercise methods in people with depression has shown benefits from both categories of exercise in reducing the depression level and depression symptoms. A comparison between the two methods was not feasible, given the limitation of designs [18]. This suggests that specifically designed studies that are rigorously conducted need to be performed comparing a mindfulness based versus standard exercise intervention to be able to discern the true and significant benefits of the mindfulness component of an intervention such as yoga. 

Another limitation of studies of yoga in breast cancer survivors is their almost exclusive appeal to the high-income, white population. The only study to include an ethnically and economically diverse population [45] reported on the effect of yoga versus usual care wait list in a multiethnic sample of 128 breast cancer survivors. There was no difference in QOL except for less decrease in social well-being in the yoga group. However, in a secondary analysis of the participants not receiving chemotherapy, significant improvements were seen in the overall QOL, emotional well-being, social and spiritual well-being, and less distressed mood in the yoga group compared to the control, wait-list group. 

Overall, yoga intervention seems to be beneficial in this population, especially in fatigued patients. However, the results of these studies should be interpreted with caution, given the small sizes and the heterogeneity of the population, outcomes and yoga intervention (duration, frequency, and type of yoga program), and methodological limitations of the studies. Similar conclusions were drawn by a systematic review of the effects of yoga on psychological outcomes in cancer survivors [81] and by a review of integrative therapies in cancer survivors [80]. 

More rigorous studies of yoga have recently been conducted capturing the attention of the scientific community. A recent clinical trial by Mustian et al. focused on yoga versus usual care in 410 cancer survivors (75% with breast cancer) with sleep disturbances. This study showed significant improvement in sleep (22% versus 12%) and fatigue (42% versus 12%) and a significant decrease in the use of sleep medications in the yoga group compared with the usual care group [85]. In an abstract presented at the annual meeting of the American Society of Clinical Oncology in 2011, Cohen et al. reported on a study of yoga versus stretching versus control wait-list group on 163 breast cancer survivors undergoing radiotherapy. Yoga and stretching were superior to the control group in improving fatigue and physical functioning. Yoga was superior to the other groups in improving QOL, benefit finding, cortisol slope, and heart rate variability [86]. From these studies and the increasing interest in yoga for cancer survivors and medical institutions, it appears that yoga is establishing itself into the mainstream management and treatment of cancer survivors.

**Tai Chi Chuan**

The studies of TCC in breast cancer are few, generally small (*N* = 16–21), poorly controlled, and have heterogeneous outcomes. Benefits were shown in improvements in QOL, fat mass, bone formation, aerobic capacity, shoulder ROM, and self-esteem. However, a recent systematic review of this intervention failed to show any benefits attributable to TCC in the four RCTs included in the review [87]. The three, small nonrandomized, controlled clinical trials included in this review did show favorable effects in psychological and physical outcomes, although the risk of bias was high.

Future studies of TCC that include a symptomatic group of breast cancer survivors and compare TCC to other forms of low-impact aerobic exercise may be useful to help understand if the effects seen from TCC are unique to this form of exercise. With the increasing recognition of the importance of metabolic abnormalities such as hyperinsulinemia [88] in the prognosis of breast cancer, and of the prevalence of long-term sequelae such as metabolic bone disease [89] in this population, rigorous studies of tai chi chuan, a low-impact exercise intervention shown to improve these outcomes in noncancer patients, should be conducted.

**Pilates**

The study of the Pilates method in breast cancer survivors is underrepresented (only three studies exist addressing this method, one being a case series [73, 74, 90]), in contrast to the intense advertising of this method in the rehabilitation of breast cancer survivors. The evidence from these studies showed improvements in aerobic capacity, QOL, mood, body image after mastectomy, and improved shoulder ROM, as well as potential concerns of lymphedema. These findings are also limited by the small size and the limited research performed in this area. No studies of Pilates in survivors of types of cancer other than breast were conducted. Strong evidence to support Pilates as an effective rehabilitation method after breast cancer treatment is lacking at this time.

**Qigong**

The four studies of qigong in breast cancer survivors all have different outcomes. A small (*N* = 9) non-controlled study reported on the effects of externally applied qigong, by a qigong master, to the cancerous mass, failing to reveal a change in size or a change in psychological outcomes. The other three studies report on the effects of qigong practiced by the participants. The largest of these studies (*N* = 162) and with the strongest methodology did show significant improvements in QOL, fatigue, mood, and CRP levels [7], but only 34% of the participants in this study had breast cancer. A recent systematic review of qigong in cancer survivors has shown a significant improvement in the immune function, but no conclusion could be drawn towards psychological outcomes, due to the heterogeneity of the outcomes and the methodological flaws [91]. At this time, not enough evidence exists to recommend the use of qigong for breast cancer patients.

## 10. Conclusion

Our review has found that studies of mindful exercise interventions in breast cancer survivors are generally small, poorly controlled, and the outcomes are heterogeneous. With the exception of evidence that yoga improves fatigue in breast cancer survivors, no other strong conclusions can be derived, given the methodological limitations of the studies. 

The significant interest by both patients and health care providers to integrate CAM therapies into the management of breast cancer survivors should hopefully lead to more effort and attention given to incorporating evidence-based CAM knowledge into clinical care. In this age of evidence-based medicine, CAM researchers will be expected to conduct RCTs that are adequately powered, well designed, and controlled, and with scrupulous attention paid to eliminating sources of bias. A multidisciplinary approach, in combination with personalized programs, has now become the state-of-the-art management of breast cancer. It is prudent that CAM researchers conducting clinical trials prioritize the need to assess safety, efficacy, and long-term benefits, while attempting to define the position of CAM in this complex therapeutic approach. With supporting evidence, health care providers are in a better position to educate survivors of breast cancer, help them make evidence-based decisions, and recommend CAM therapies that are demonstrated to improve QOL of their patients. 

## Figures and Tables

**Table 1:** Summary of studies involving yoga interventions in breast cancer survivors (in order of the publication date).

Reference	Intervention (type/duration)	Study design	*N* and characteristics	Main outcomes	Results/comments (group by time interactions reported for the controlled studies and time effects for noncontrolled studies)
Galantino et al. [24], 2012	Hatha yoga 10 weeks, 90′ sessions, 2x/week	One-arm qualitative, exploratory design	10 Aromatase inhibitors associated arthralgias	Performance accomplishment Structured experience Verbal support Physical feedback	Themes discovered: (i) empowerment (importance of camaraderie, community, and sharing) (ii) pain relief (iii) increased physical fitness (energy, flexibility, and function); relieved stress/anxiety (iv) transferability of yoga through breathing

Bower et al. [17], 2011	Iyengar yoga versus health education 12 weeks, 90′ sessions, 2x/week	RCT	31 Persistent fatigue Stages I-II Postmenopausal	Fatigue (FSI) Vigor (MFSI) Depression (BDI-II) Sleep (PSQI)	Decreased fatigue* Increased vitality* Increased vigor* More confident on managing fatigue* Decreased depressive symptoms* No difference in sleep

Galantino et al. [9], 2011	8 weeks, twice a week	One arm	10 Postmenopausal with AIs-induced arthralgias	Balance (Functional reach) Flexibility (Sit and Reach) Pain (BPI) Function (PSFS) QOL (FACT-B)	**Improvements in balance, flexibility, function, pain severity, and QOL** Trend towards reduced pain interference 80% adherence to the home program

Banasik et al. [25], 2011	Iyengar yoga versus wait list 8 weeks, 90′ sessions, 2x/week	RCT	18 Stage II-IV	QOL (FACT-B) Fatigue Likert scale Salivary cortisol	No difference in QOL Decreased fatigue* No difference in the slope of cortisol

T. Kovačič and M. Kovačič [26, 27], 2011	Yoga in daily life system + PT versus PT 4 weeks	RCT	32 Immediately after surgery	Self-esteem (RSES) General health (GHQ-12) Symptoms (RSCL) Stress (PSS)	Improved self-esteem* Less distress during hospitalization and afterwards*

Littman et al. [28], 2011	Viniyoga (at home or classes) versus wait list 5x/week for 6 months	RCT	63 Obese and overweight women (BMI ≥ 24)	Feasibility (time to recruit, retention, adherence) QOL (FACT-G, FACT-B) Fatigue (FACIT-F) Weight and height Waist and hip circumference	12 months to recruit Attendance was 20 classes and 56 at home practices in 6 months 51% were satisfied the program Trend towards improved QOL and fatigue **Decreased waist circumference by** −3.1cm* No change in weight, BMI, and hip circumference

Bower et al. [29], 2011	Iyengar yoga 12 weeks, 90′ sessions, twice weekly	One arm	12 Persistent fatigue	Fatigue (FSI) Depression (BDI-II) Sleep (PSQI) Pain (BCPTSS) QOL (SF-36) Physical function (8-foot walk test, chair stands) Program Evaluation	Decreased fatigue and number of days with fatigue/week* Improved vitality, depression, and general health* No difference in sleep Trend towards decreased pain All improvements persisted at 3 months after intervention* Improvement in physical function* High satisfaction with the program

Desai et al. [30], 2010	Any type of yoga	Survey of yoga use	300 Users of AIs	Sociodemographics of yoga users	17.7% breast cancer survivors used versus 6% in general population Yoga use associated with white race, lower BMI, higher education, higher socioeconomic status, part-time employment, stage II cancer,previous chemotherapy, and previous radiotherapy* In multivariate analysis, yoga use was associated with higher education and lower BMI*

Speed-Andrews et al. [31], 2010	Iyengar yoga 12 week classes	One arm	24	QOL (SF-36, FACT-B) Fatigue (FSI) Stress (PSS) Anxiety (STAI) Depression (CESSDS) Body image (brief body image scale) Self-esteem (Rosenberg Self-Esteem Scale) Happiness (the happiness measure) Motivational outcomes Program evaluation	Improved generic QOL (mental health, vitality, pain, and roleemotional)* Trend of improvement in breast specific-QOL Trend of improvement on stress, depression, body image, and self-esteem Strong motivational response Very high satisfaction with the program, very high perceived benefit

Ülger and Yağli [32], 2010	8 yoga sessions	One arm	20	QOL (NHP) Stress (STAI-I, STAI-II) Satisfaction with the program	Improved QOL* Decreased anxiety* High satisfaction with yoga program

Chandwani et al. [33], 2010	yoga versus wait list 6 weeks 2x/week	RCT	61 Undergoing radiotherapy	QOL Fatigue Meaning finding Intrusive thoughts Sleep Depression/anxiety	Improved health perception, physical functioning scores, more intrusive thoughts, and greater meaning finding* No difference in fatigue, depression, sleep

Vadiraja et al. [34], 2009	Integrated yoga program (18–24, 60′ sessions) plus brief supportive therapy (every 10 days) versus brief supportive therapy (every 10 days)	RCT	88 Stage II-III Undergoing radiotherapy	QOL (EORTCQOL C30) functional scales Affect (PANAS)	Improved positive affect* Improved emotional function* Improved cognitive function* Decrease in negative affect* **Positive correlation between positive affect and physical, emotional, cognitive, and social function and global QOL** ^∗ ^

Carson et al. [35], 2009	Yoga of awareness versus wait list 8 weeks	RCT	37 Stage I-II Vasomotor symptoms	Hot flashes before, after, and at 3 months after intervention	Decreased hot flash frequency, severity, and total score* Improved joint pain, fatigue, sleep, bother, vigor, negative mood* **(maintained at 3 months) ** **More time practicing positively correlated with less fatigue, less bother, and more acceptance** ^∗ ^

Vadiraja et al. [36], 2009	Integrated yoga program (18–24, 60′ sessions) plus brief supportive therapy (every 10 days) versus brief supportive therapy (every 10 days)	RCT	88 Stage II-III Undergoing radiotherapy	6 AM salivary cortisol level before and after radiotherapy Self-rated anxiety, depression, and stress before and after radiation therapy	Significant decreased anxiety, depression, perceived stress, and salivary cortisol* **Cortisol level positively correlates with anxiety and depression** ^∗ ^

Vadiraja et al. [37], 2009	Integrated yoga program (18–24, 60′ sessions) plus brief supportive therapy (every 10 days) versus brief supportive therapy (every 10 days)	RCT	88 Stage II-III Undergoing radiotherapy Mastectomy	Symptoms (RSCL) QOL (EORTCQOL C30) symptom scale	Decreased fatigue* Decreased insomnia* Decreased appetite loss* Decreased psychological distress* No change in physical distress No change inactivity level **Distress positively correlated with fatigue, nausea, vomiting, pain, dyspnea, insomnia, appetite loss, and constipation**

Danhauer et al. [38], 2009	Restorative yoga versus wait list Weekly 75′ sessions × 10 weeks	RCT	44 34% in active treatment	Physical Health (SF-12) QOL (FACT-B) Fatigue (FACT-Fatigue) Spiritual well-being (FACIT-Sp) Depression (CES-D) Sleep (PSQI) Affect (PANAS) Feasibility Program Evaluation	Improved mental health, depression, positive affect, and spirituality* Greatest benefit on participants with higher negative affect and lower emotional well-being at baseline* Trend towards decreased sleep latency and increased QOL Recruitment 19%, adherence 58%- higher in women with higher baseline physical health and QOL High satisfaction with class, no adverse events

Rao et al. [39], 2009	Integrated yoga program (1–7 weekly 60′ sessions for 24 weeks) plus 3-4 brief supportive therapy every 10 days versus brief supportive therapy every 10 days	RCT	98 Stage II-III Radiotherapy Chemotherapy	Anxiety (STAI) Symptom checklist	Decreased anxiety andsymptom severity* **Anxiety states positively correlate with symptoms severity and distress** ^∗ ^

Rao et al. [40, 41], 2008	Integrated yoga program versus supportive therapy + exercise rehabilitation 4 weeks	RCT	98 Stage II-III Immediately at diagnosis	Anxiety (STAI) Depression (BDI) QOL (FLIC) Symptom checklist Lymphocytes, Immunoglobulins Cytokines Hospital stay Drain retention Time to suture removal Postoperative complications	Decreased anxiety, depression, and treatment-related symptoms after surgery* **Increased QOL after surgery** Less decrease in CD56% after surgery* Decrease in IgA levels after surgery* Significant decrease in hospital stay, drain retention, days to suture removal* Decreased TNF*α*after surgery*

Danhauer et al. [42], 2008	Restorative yoga Weekly 75′ sessions × 10 weeks	One-arm pilot	51 Breast and ovarian cancer (*N* = 14 with breast cancer)	Physical Health (SF-12) QOL (FACT-G) Spiritual well-being (FACIT-Sp) Fatigue (FACT-Fatigue) Depression (CES-D) Anxiety (STAI) Affect (PANAS) Feasibility Program evaluation	Improvedmental health, QOL, fatigue, depression, state anxiety, and negative effect* No change in positive affect and spiritual well-being Better adherence was associated with better physical health High satisfaction with the program (88% positive)

Rao et al. [43], 2008	Integrated yoga versus brief supportive therapy	RCT	37 Stage II-III Active cancer treatments	NK cell % after surgery, radiation, and chemo	NK cell% was higher after chemo* No difference in NK percentage after surgery and after chemo

Raghavendra et al. [44], 2007	Integrated yoga by instructor (at chemo and every 10 days and at home 60′ daily) versus supportive therapy (30–60′ at chemo and every 10 days)	RCT	62 Stage II-III Postmastectomy Post radiation Undergoing chemotherapy	Nausea and emesis (MANE) Anxiety (STAI) Depression (BDI) QOL (FLIC) Symptom check list Treatment-related toxicity and side-effects (WHO Toxicity criteria)	Reduced frequency and intensity of chemo-associated nausea* Trend towards reduced frequency and intensity of chemo-associated vomiting* Reduced intensity and frequency of anticipatory nausea and vomiting* Nausea and vomiting (both anticipatory and after chemo), positively correlated with anxiety, depression, distress, and chemo-related toxicity and negatively with QOL* Decreased anxiety, depression, and distress* Increased QOL* **Decreased treatment toxicity** ^∗ ∗ ^

Moadel et al. [45], 2007	Hatha yoga versus wait list 12 weekly −90′ sessions	RCT	128 Ethnically diverse	QOL (FACT-B, FACT-G) Fatigue (FACIT-F) Spirituality (FACIT-Sp) Depressed mood Index mood (POMS) Adherence Program evaluation	Less decrease in social well-being* Subgroup analysis for nonchemo patients: improved QOL, emotional, social, and spiritual well-being, distressed mood, anxiety, and irritability* Adherence was positively associated with physical well-being and negatively associated with fatigue and distressed mood* Breathing and meditation components were rated higher than the social connection

Banerjee et al. [46], 2007	Integrated yoga versus supportive counseling plus light exercise 90′ sessions for 6 weeks, frequency not specified	RCT	68 Radiation therapy	Anxiety/depression (HADS) Stress (PSS) DNA damage assay	**Decreased anxiety, depression perceived stress, and DNA damage** ^∗ ^

Carson et al. [47], 2007	Yoga of awareness Weekly sessions for 8 weeks	One arm	21 Metastatic disease	Daily measures of pain, fatigue, distress, invigoration, acceptance, and relaxation Focus group feedback Focus Group Questionnaire	Increase in daily invigoration and acceptance* Trend towards improvements in pain and relaxation Greater yoga practice positively associated with decreased pain, increased invigoration, and acceptance* Greater yoga practice positively associated with decreased next-day pain and fatigue and increased invigoration, relaxation, and acceptance* Program was considered overall very helpful

Culos-Reed et al. [48], 2006	Modified Hatha yoga versus wait list 7 weeks of weekly 75′ sessions	RCT	38	Mood (POMS) Response to stress (SOSI) QOL (EORTC QLQ-C30) Physical activity (LSI) Fitness (CPA-FLA)	Improvements in QOL, emotional functioning, and diarrhea* Trend toward improved emotional irritability, gastrointestinal symptoms, cognitive disorganization, mood, tension, depression, and confusion No difference in physical activity and fitness

AIs: Aromatase Inhibitors; BCPTSS: Breast Cancer Symptom Scale; BDS: Beck Depression Scale; BDI: Beck Depression Inventory; BPI: Brief Pain Inventory; CAM: Complementary and Alternative Medicine; CES-D: Center for Epidemiologic Studies-Depression Scale; CPA-FLA: Canadian Physical Activity, Fitness and Lifestyle Appraisal; EORTC QLQ-C30: European Organization for Research and Treatment Core Quality of Life Questionnaire-C30; FACT-B: Functional Assessment of Cancer Therapies-Breast; FACT-G: Functional Assessment of Cancer Therapies-General; FACIT-F: Functional Assessment of Chronic Illness Therapy-Fatigue; FACIT-Sp: Functional Assessment of Chronic Illness Therapy-Spirituality; FLIC: Functional Living Index for Cancer; FSI: Fatigue Symptom Inventory; GHQ-12: General Health Questionnaire-12; HADS: Hospital Anxiety and Depression Scale; IL-2R: Interleukin 2 Receptor; INF: Interferon; LOT-R: Life Orientation Test-Revised; LSI: Leisure Score Index; MANE: Morrow Assessment of Nausea and Emesis; MFSI: Multidimensional Fatigue Symptom Inventory; NHP: Nottingham Health Profile; PANAS: Positive and Negative Affect Schedule; POMS: Profile of Mood States; PSFS: Patient-Specific Functional Scale; PSQI: Pittsburgh Sleep Quality Inventory; PSS: Perceived Stress Scale; RCT: randomized controlled study (yoga versus control group); QOL: Quality of Life; RSCL: Rotterdam Symptom Checklist; RSES: Rosenberg; Self-Esteem Scale; SF-12: Short Form-12 Health Survey; SF-36: Medical Outcome Studies Short Form; SOSI: Symptoms of Stress Inventory; STAI: State Trait Anxiety Inventory; TNF: Tumor Necrosis Factor; bold text with^∗  ^: statistically significant (*P* < 0.05).

**Table 2:** Studies involving tai chi chuan in breast cancer survivors.

Reference	Intervention (type/duration)	Study design	*N*	Main outcomes	Comments/results (group by time interactions reported for the controlled studies and time effects for non-controlled studies)
Reid-Arndt et al. [65], 2012	Yang style tai chi chuan 10 weeks, 60′ sessions, 2x/week	One arm	23 (16 with breast cancer) At least 12 months from chemotherapy	Neuropsychological tests (memory, executive function, language, and attention) Self-reported cognitive functioning (MASQ) Distress (IES-R) Mood (POMS-SF) Fatigue (POMS-SF) Balance	Improvements in immediate and delayed memory, verbal fluency, attention, executive functioning, and self-reported cognitive functioning* Improvements in stress* Trend toward improved vigor No changes in fatigue Improved balance*

Sprod et al. [66], 2012	Yang style tai chi chuan versus standard support therapy 12 weeks, 60′ sessions, 3x/week	RCT	35	HRQOL (MOS SF-36) IL-6, IL-8 Glucose Cortisol Insulin, IGF-1; IGFBP-1; IGFBP-3	Improved physical functioning and general mental health* Trends towards improved social functioning and lack of increase in insulin levels

Janelsins et al. [67], 2011	Yang style tai chi chuan versus psychosocial support therapy 12 weeks, 60′ sessions, 3x/week	RCT	19	Insulin, IGF-I, IGFBP Body composition (weight, bmi, fat mass, fat-free mass) Cytokine levels (IL-6, IL-2 and IFN-*γ*	Lack on increase in insulin levels* No change in IGF-1, IGFBP or cytokines Decreased BMI*

Peppone et al. [68], 2010	Yang style tai chi chuan versus psychosocial support therapy 12 weeks, 60′ sessions, 3x/week	RCT	16	Bone formation (serum BSAP) Bone resorption (serum NTx) Bone remodeling index IGF-1, IGFBP1,3 Cytokines (IL-2, 6, 8, IFN-*γ*2)	Trend towards an increase in bone formation and a decrease in bone resorption Improvement in bone remodeling index*

Mustian et al. [69–71], 2008, 2006, 2004	Yang style tai chi chuan versus psychosocial support therapy 12 weeks, 60′ sessions, 3x/week	RCT	21	Functional capacity (aerobic capacity, muscle strength, and flexibility) QOL (FACIT-Fatigue) Body composition Self-esteem (RSE)	Trend toward improvement in aerobic capacity and flexibility Improved muscle strength and QOL at 12 weeks* No difference in body composition Improved self-esteem* Self-esteem positively correlated with QOL*

BSAP: Bone-Specific Alkaline Phosphatase; FACIT-Fatigue: Functional Assessment of Chronic Illness Therapy-Fatigue; HRQOL: health-Related Quality of Life; IGFBP: Insulin-like Growth Factor Binding Protein; IL: Interleukin; IGF: Insulin-like Growth Factor; MASQ: Multiple Abilities Self-Report Questionnaire; NTx: N-Telopeptides of Type I Collagen; IES-R: Impact of Event Scale-Revised; POMS-SF: Profile of Mood States-Short Form; RSE: Rosenberg Self-Esteem Scale; bold text with*: statistically significant (*P* < 0.05).

**Table 3:** Studies involving Pilates method in breast cancer survivors.

Reference	Intervention (type/duration)	Study design	*N*	Main outcomes	Comments/results (group by time interactions reported for the controlled studies and time effects for non-controlled studies)
Stan et al. [73], 2012	Mat Pilates 12 weeks, 45′ sessions, 3–5x/week Postmastectomy	One arm	15 All had mastectomy	Shoulder ROM Spine flexibility Height Arm volumes QOL (FACT-B) Mood (POMS) Body image (MBSRQ)	Improved shoulder abduction and internal rotation* Improved neck flexion and rotation towards the unaffected side* No difference in spine flexibility and height Increased arm volume of the affected compared to the unaffected side (subclinical lymphedema in 6 patients)* Improved QOL and certain scales of mood and body image*

Eyigor et al. [74], 2010	Mat Pilates plus home exercise (walking, stretching, and ROM) versus home exercises 8 weeks, 60′ sessions, 3x/week	RCT	52 All had mastectomy	Aerobic capacity (6MWT) Flexibility (modified sit and reach test) Fatigue (BFI) Depression (BDI) QOL (EORTC-QLQ-C30 and B23)	Improved aerobic capacity* No difference in the other outcomes

6MWT: 6-Minute Walk Test; BFI: Brief fatigue Inventory; BDI: Beck Depression Index; BPI: Brief Pain Inventory; EORTC-QLQ-C30: European Organization for Research and Treatment Cancer-Quality of Life; FACT-B: Functional Assessment of Cancer Therapies-Breast; MBSRQ: Multidimensional Body-Self-Relations Questionnaire; POMS: Profile of Mood States-Short Form; UE: Upper Extremity; bold text with*: statistically significant (*P* < 0.05).

**Table 4:** Studies involving qigong in breast cancer survivors.

Reference	Intervention (type/duration)	Study design	*N*	Main outcomes	Comments/results (group by time interactions reported for the controlled studies and time effects for non-controlled studies)
Cohen et al. [77], 2010	External qigong (applied by qigong master) daily (2–5′) for 5 consecutive days	One arm	9 Untreated cancer Tumor size ≤ 3 cm	Tumor size by breast imaging QOL (FACT-B) Distress (BSI) Cancer-related symptoms (MIDAS)	No difference in tumor size No difference in QOL, distress, and symptoms

Oh et al. [7], 2010	Medical qigong versus usual care 10 weeks, 90′ sessions 2x/week Home practice 30′ daily	RCT	162 Only 34% were breast cancer survivors	QOL (FACT-G) Fatigue (FACT-Fatigue) Mood (POMS) Inflammation (CRP)	Improvements in all domains of QOL* Improvement in fatigue* **Improvement in overall mood and all subscales of mood, except for anger-hostility and confusion subscale** Improvement in CRP level*

Yeh et al. [78], 2006	Chan-Chuang qigong versus no intervention 21 days	CCT	67 Undergoing chemotherapy	CBC (on days 0, 8, 15, and 22 days of chemotherapy)	No change in WBC, platelets, and hemoglobin Better rebound of WBC after 21 days*

Lee et al. [79], 2006	Chan-Chuang qigong versus no intervention 21 days	CCT	67 Undergoing chemotherapy	Symptom distress (SDS) Psychological distress (SCL-90-R) (on days 0, 8, 15, and 22 days of chemotherapy)	Improved overall symptom score at day 22* Less numbness and heartburn on day 8, less pain and numbness on day 15, less pain, numbness, heartburn, and dizziness on day 22* No difference in overall psychological score Less hopelessness about the future on day 8, less unwillingness to live on day 22*

BSI: Brief Symptom Inventory; CRP: C-Reactive Protein; CBC: Complete Blood Count; FACT-B: Functional Assessment of Cancer Therapies-Breast; FACT-F: Functional Assessment of Cancer Therapies-Fatigue; POMSs: Profile of Mood States; MDASI: MD Anderson Symptom Inventory; SCL-90-R: Symptom Checklist-Revised; SDS: Symptom Distress Scale; bold text with*: statistically significant (*P* < 0.05).
